# Longitudinal associations between neighbourhood walkability and incident childhood asthma

**DOI:** 10.1186/1710-1492-10-S1-A8

**Published:** 2014-03-03

**Authors:** Elinor Simons, Sharon Dell, Rahim Moineddin, Teresa To

**Affiliations:** 1 Clinical Epidemiology, Department of Health Policy, Management and Evaluation, University of Toronto, Toronto, ON, Canada; 2 Child Health Evaluative Sciences, Hospital for Sick Children, Toronto, ON, Canada, M5G 1X8; 3 Respiratory Medicine, Hospital for Sick Children, Toronto, ON, Canada, M5G 1X8; 4 Department of Family and Community Medicine, University of Toronto, Toronto, ON, Canada, M5G 1V7

## Background

Low neighbourhood walkability has been associated with chronic conditions such as obesity and diabetes, but the relationship between neighbourhood walkability and incident childhood asthma has not been determined.

## Methods

We evaluated the association between neighbourhood walkability and incident childhood asthma using prospectively-collected administrative data at the Institute for Clinical Evaluative Sciences. Among the 1997-2003 birth cohorts of children living in Toronto, neighbourhood quintile of walkability was reported using a validated walkability index with 4 dimensions: population density, dwelling density, access to services and street connectivity. Incident asthma was defined by the time of entry into the Ontario Asthma Surveillance Information System (OASIS) database, requiring 2 outpatient visits for asthma within 2 consecutive years or any hospitalization for asthma. Sex and neighbourhood income quintile were obtained from the Registered Person’s Database. Histories of preterm delivery, obesity and other atopic conditions were obtained from Ontario Health Insurance Plan records. We calculated the associations between incident childhood asthma and the two lowest versus the two highest neighbourhood walkability quintiles using Cox proportional and discrete-time hazard models.

## Results

Twenty-one percent of the 326 383 children met the OASIS criteria for asthma. After adjusting for sex, preterm delivery, obesity, atopic conditions and neighbourhood income quintile, children with low home neighbourhood walkability at birth were at increased risk of asthma development [hazard ratio (HR) 1.11; 95% confidence interval (CI), 1.08-1.14], and the association did not change for children with healthcare visits for asthma in the past year (HR 1.10; 95% CI, 1.04-1.16). When walkability in each year of the child’s life was considered, low neighbourhood walkability was associated with increased odds of incident childhood asthma (odds ratio 1.12; 95% CI, 1.09-1.15). These associations were not substantially affected by year of birth within the cohort.

## Conclusions

Children living in neighbourhoods with low walkability are at increased risk of incident childhood asthma after adjusting for neighbourhood and individual characteristics. The association persists for children with visits for asthma within the past year. Possible mechanisms of this association include more physical activity, fewer weight problems and decreased exposure to traffic-related air pollution in more walkable neighbourhoods. Our findings suggest that promotion of neighbourhood walkability may offer a strategy for primary asthma prevention. Walkability of existing neighbourhoods may be improved by encouraging greater placement of services such as banks and grocery stores within walking distance of residential neighbourhoods and adding pedestrian paths between roads to improve street connectivity.

